# Aquaporin-4 Autoantibodies From Neuromyelitis Optica Spectrum Disorder Patients Induce Complement-Independent Immunopathologies in Mice

**DOI:** 10.3389/fimmu.2018.01438

**Published:** 2018-06-25

**Authors:** Leung-Wah Yick, Oscar Ka-Fai Ma, Roy Chun-Laam Ng, Jason Shing-Cheong Kwan, Koon-Ho Chan

**Affiliations:** ^1^Department of Medicine, Li Ka Shing Faculty of Medicine, The University of Hong Kong, Hong Kong; ^2^Neuroimmunology and Neuroinflammation Research Laboratory, Li Ka Shing Faculty of Medicine, The University of Hong Kong, Hong Kong, Hong Kong

**Keywords:** aquaporin-4, astrocyte, autoimmunity, axonal damage, demyelination, microglia, neuroinflammation, neuromyelitis optica

## Abstract

Neuromyelitis optica spectrum disorders (NMOSD) are central nervous system inflammatory disorders causing significant morbidities and mortality. The majority of NMOSD patients have autoimmunity against aquaporin-4 (AQP4), evidenced by seropositivity for autoantibodies against aquaporin-4 (AQP4–IgG). AQP4–IgG is pathogenic with neuroinflammation initiated upon binding of AQP4–IgG to astrocytic AQP4. Complement activation contributes to astrocytic cytotoxicity, neuroinflammation, and tissue necrosis in NMOSD, but the role of complement-independent mechanisms is uncertain. We studied the complement-independent pathogenic effects of AQP4–IgG by passive transfer of IgG from NMOSD patients to mice with breached blood–brain barrier (BBB). Mice, pretreated with bacterial proteins, received daily intraperitoneal injections of IgG purified from AQP4–IgG-seropositive NMOSD patients [IgG_(AQP4+)_], or IgG from AQP4–IgG-seronegative patients [IgG_(AQP4−)_] or healthy subjects [IgG_(Healthy)_] for 8 days. Motor function was tested by walking across narrow beams, and spinal cords were collected for immunofluorescent analysis. We found that human IgG infiltrated into cord parenchyma of mice with breached BBB without deposition of complement activation products. Spinal cord of mice that received IgG_(AQP4+)_ demonstrated loss of AQP4 and glial fibrillary acidic protein (suggestive of astrocyte loss), decrease in excitatory amino acid transporter 2, microglial/macrophage activation, neutrophil infiltration, patchy demyelination, and loss in axonal integrity. Mice that received IgG_(AQP4+)_ required longer time with more paw slips to walk across narrow beams indicative of motor slowing and incoordination. Our findings suggest that AQP4–IgG induces complement-independent cord pathologies, including astrocytopathy, neuroinflammation, demyelination, and axonal injuries/loss, which are associated with subtle motor impairments. These complement-independent pathophysiologies likely contribute to early NMOSD lesion development.

## Introduction

Neuromyelitis optica spectrum disorders (NMOSD) are central nervous system (CNS) inflammatory demyelinating disorders (CNS IDD) characterized by recurrent episodes of acute optic neuritis, myelitis, and less frequently encephalitis. Severe NMOSD attacks, typically bilateral optic neuritis and longitudinally extensive transverse myelitis can lead to permanent blindness, paraplegia, and even mortality (1). Neuromyelitis optica immunoglobulin G was discovered in 2004 as a serum autoantibody biomarker of neuromyelitis optica (2), and subsequently confirmed to be IgG autoantibodies targeting aquaporin-4 (AQP4–IgG) (3). Aquaporin-4 (AQP4) is the most abundant water channel in the mammalian CNS. It is highly expressed in the membrane of astrocytic endfeet at the interfaces between blood/cerebrospinal fluid and brain parenchyma. AQP4 is vital for water homeostasis of the CNS. Current evidence supports that AQP4–IgG is pathogenic and neuroinflammation is initiated upon binding of AQP4–IgG to astrocytic AQP4. *In vitro* studies suggest that binding of polyclonal AQP4–IgG to different epitopes of AQP4 triggers various outcomes which include AQP4 internalization and endolysosomal degradation (4), inflammatory cell infiltration (5), impairment of glutamate uptake (6, 7) and water flux (8), and breakdown of the blood–brain barrier (BBB) (9).

Previous *in vivo* studies on NMOSD pathophysiologies focus on complement-dependent mechanisms triggered predominantly by autoantibody–AQP4 interaction (10–14). However, the initiator of complement cascade, C1q, is absent in the quiescent CNS. Primary binding of AQP4–IgG to astrocytic AQP4 does not trigger complement activation immediately. This suggests that complement-dependent mechanisms, which require local complement synthesis or complement entry from peripheral blood, may occur relatively late in acute attacks of NMOSD (15, 16). The *in vivo* complement-independent pathological effects of AQP4–IgG and their role in NMOSD lesion development are unclear. Indeed, a wide spectrum of pathologies with six different lesion types are reported in NMOSD patients suggesting that acute attacks involve complex and multiple mechanisms of tissue injury including both complement-dependent and complement-independent mechanisms (17).

As human IgG does not activate mouse complements (18), we previously studied the complement-independent pathological effects of AQP4–IgG by passive transfer intraperitoneally to mice with BBB breached by prior immunization with bacterial proteins before transfer of human IgG (19). We reported that spinal cord of mice which received IgG isolated from sera of AQP4–IgG-seropositive patients had AQP4 loss and astrocytic activation (not observed in mice which received IgG from healthy subjects or AQP4–IgG-seronegtive patients), but no glia fibrillary acidic protein (GFAP) loss, inflammatory cell infiltration, demyelination, or any clinical weakness documented by the experimental autoimmune encephalomyelitis (EAE) score (19). However, this may be related to the low dose of IgG transferred (2 mg daily for 3 days, total 6 mg in each mouse). The current study aimed to investigate (1) the complement-independent pathological effects of AQP4–IgG on glial and immune cells in the spinal cord of mice with breached BBB at a higher dose of human IgG transferred (4 mg daily for 8 days, total 32 mg in each mouse) and (2) whether the AQP4–IgG-induced pathologies on glial and immune cells are associated with neuronal injuries and subtle motor impairments not documented by the EAE score. We observed that mice which received IgG from AQP4–IgG-seropositive NMOSD patients developed significant AQP4 and astrocyte loss, excitatory amino acid transporter 2 (EAAT2) decrease, microglial/macrophage activation, neutrophil infiltration, demyelination, and axonal injuries/loss in the spinal cord compared to controls; and these cord pathologies were associated with subtle motor impairments.

## Materials and Methods

### Patients and Control Subjects

Sera/plasma were obtained from (1) 13 NMOSD patients seropositive for AQP4–IgG detected by a cell-based indirect immunofluorescence assay as described previously (20), (2) 5 NMOSD patient seronegative for both AQP4–IgG and myelin-oligodendrocyte glycoprotein (MOG)-IgG by cell-based assays, and (3) 3 healthy subjects. Informed consent for study was obtained from all study subjects.

### IgG Purification

IgG was purified from sera or plasma using the HiTrap Protein G Sepharose columns (GE Healthcare Bio-sciences, USA). The samples were further dialyzed with Slide-A-Lyzer Dialysis Cassettes (Thermo Scientific, USA) and concentrated with Amicon Ultra-15 centrifugal filters (Merck Millipore, Germany). Protein concentration was measured by Bradford assay (Bio-Rad, USA). For immunoblotting, proteins (5 µg per lane) were separated on 10% SDS polyacrylamide gels, transferred onto polyvinylidene fluoride membrane, detected using peroxidase-conjugated rabbit anti-human IgG (1:10,000, Dako, Denmark) and visualized using chemiluminescence (Advansta, USA). Pooled IgG isolated from AQP4–IgG-seropositive NMOSD patients were termed IgG_(AQP4+)_, pooled IgG isolated from AQP4–IgG- and MOG-IgG-seronegative NMOSD patients were termed IgG_(AQP4−)_, and pooled IgG isolated from healthy subjects were termed IgG_(Healthy)_.

### Breakdown of BBB and Passive Transfer of Human IgG to Mice

Female wild-type C57BL/6 mice of age 6–8 weeks were used. Mice were housed in the animal facilities at the Laboratory Animal Unit of The University of Hong Kong. They were kept in groups of five per cage under a 12 h dark/light cycle and provided with free access water and chow. All procedures were approved by the Committee on the Use of Live Animals in Teaching and Research of The University of Hong Kong.

**Figure 1:**
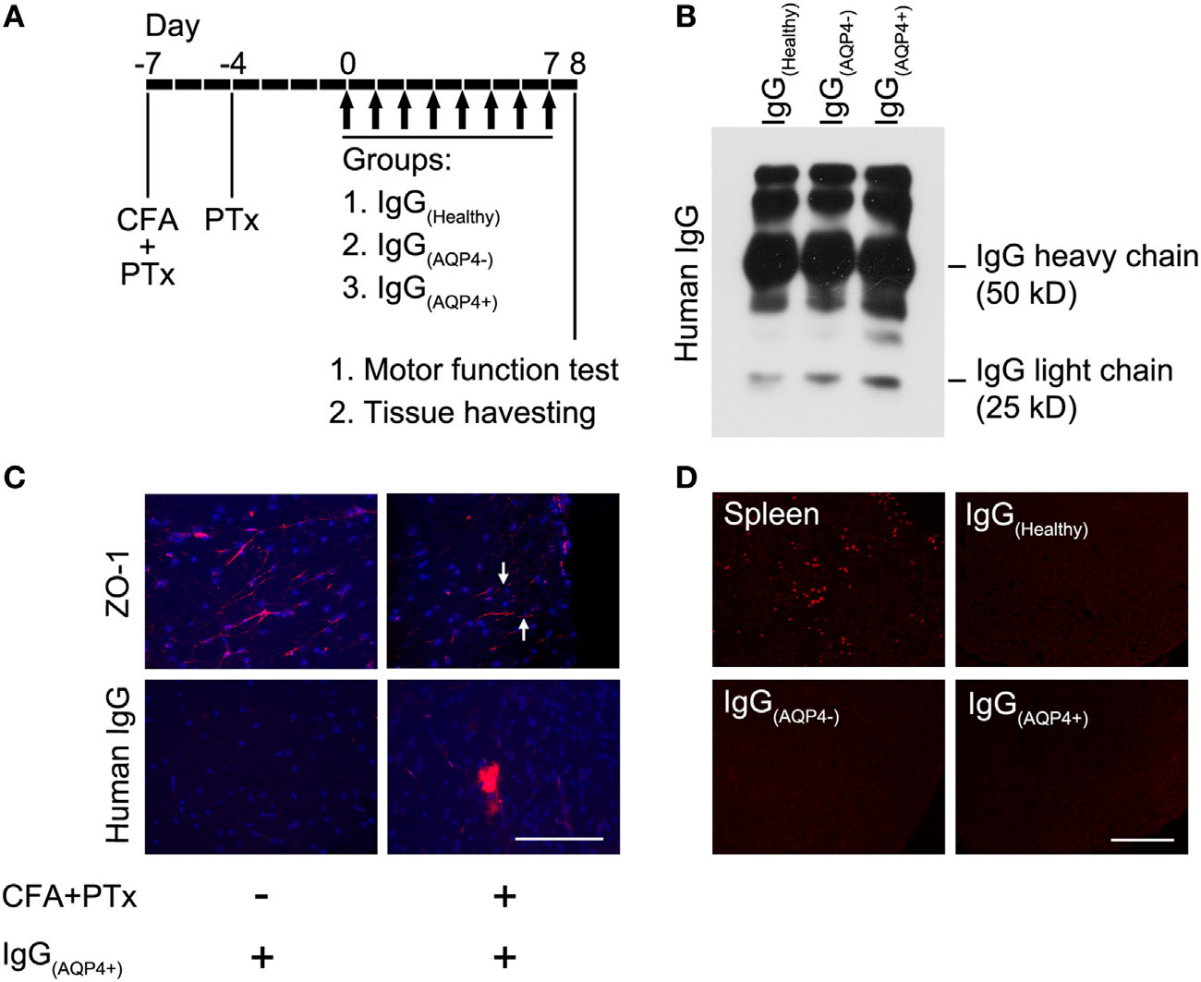
Human IgG infiltrates to the mouse spinal cord without complement activation after blood–brain barrier (BBB) breaching. **(A)** Seven days before IgG transfer, mice were injected with CFA and pertussis toxin (PTx) to breach the BBB, a second dose of PTx was injected 4 days prior to IgG transfer. From day 0 to 7, mice received daily intraperitoneal injection of IgG purified from sera/plasma of AQP4–IgG-seropositive neuromyelitis optica spectrum disorders (NMOSD) patients [IgG_(AQP4+)_], or IgG purified from sera/plasma of AQP4–IgG-seronegative NMOSD patients [IgG_(AQP4−)_] or healthy individuals [IgG_(Healthy)_] (controls). Each mouse received 4 mg of human IgG per day for 8 days, totally 32 mg human IgG. **(B)** Validation of purified IgG_(AQP4+)_, IgG_(AQP4−)_, and IgG_(Healthy)_ with heavy and light chains on Western blot analysis. **(C)** Immunofluorescence revealed discontinuous staining of the tight junction protein, ZO-1, in blood vessel of spinal cord of mice treated with CFA and PTx (arrows, upper panel). Immunofluorescence for human IgG showed infiltration of human IgG into spinal cord parenchyma after BBB breaching with CFA and PTx pretreatment, compared to control without CFA and PTx pretreatment (lower panel). **(D)** Immunofluorescence for C5b-9 revealed absence of the complement activation product in spinal cord of mice after transfer of human IgG, compared to the spleen of a mouse with experimental autoimmune encephalomyelitis induced by immunization with myelin-oligodendrocyte glycoprotein as a positive control. Scale bars = 100 µm.

Procedures of the animal experiments were summarized in Figure 1A. The BBB was breached as described by our group previously (19). In brief, mice were anesthetized with ketamine (100 mg/kg) and xylazine (10 mg/kg) and then received subcutaneous injection of complete Freund’s adjuvant (CFA, Sigma-Aldrich, USA) containing heat-killed H37Ra *Mycobacterium tuberculosis* (Difco, USA) at 4 sites, 50 µg in 50 µl at each site, on the hind flank 7 days before IgG transfer. In addition, mice received two intraperitoneal (i.p.) injections of pertussis toxin (PTx, List Biological Laboratories, USA), 200 ng in 0.2 ml PBS each, 7 and 3 days before IgG transfer. At day 0, animals received daily i.p. injection of purified human IgG for 8 consecutive days (4.0 mg IgG in 0.5 ml PBS daily, total 32 mg IgG per mouse). A total of 35 mice were used, in which 8 mice received each of PBS, IgG_(Healthy)_, IgG_(AQP4−)_, and IgG_(AQP4+)_. The remaining three mice received IgG_(Healthy)_ without CFA and PTx pretreatment as the control for BBB breakdown. We designated IgG_(AQP4+)_ mice as mice which had received IgG_(AQP4+)_, IgG_(AQP4−)_ mice as those which had received IgG_(AQP4−)_ and IgG_(Healthy)_ mice as those which had received IgG_(Healthy)_.

### Monitoring of Clinical Signs of Encephalomyelitis and Detection of Subtle Motor Abnormalities

Motor weakness of mice was assessed by EAE score as previously described (19, 21). Mice were weighted and examined daily, since day 0 till sacrifice on a 6-grade scale: 0 = no clinical signs; 1 = weight loss, limp tip of tail; 2 = limp tail, mild paraparesis; 3 = moderate paraparesis, ataxia; 4 = tetraparesis; 5 = moribund. Mice would be killed if they had weight loss exceeding 20–30% of initial body weight or developed severe weakness (score 4–5).

Subtle motor abnormalities were detected by the beam walking test which examined the animal’s ability to keep upright and walk across an elevated narrow beam to a platform (22, 23). The apparatus consisted of 1-m long beams with width of 12 or 6 mm, resting 50 cm above a table top on two stands. Prior to CFA and PTx treatment and IgG transfer, mice were trained for 2 days, three trials a day, with walking across each of the 12 and 6 mm-wide beams. One day after completion of IgG transfer, the time for the mice to cross each beam and the number of paw slips during the process was recorded.

### Tissue Preparation and Immunohistochemistry (IHC)

Mice were sacrificed after beam walking test by pentobarbital overdose and received intracardiac perfusion with ice-cold PBS and paraformaldehyde. Cervical spinal cords were harvested, and sectioned in 10 µm with a cryostat. IHC was performed with standard procedures. Sections were incubated with the following primary antibodies at 4°C overnight: (1) rabbit anti-human IgG (1:600, Dako), (2) rabbit anti-complement membrane attack complex (C5b-9, 1:200, Abcam), (3) rabbit anti-zonula occludens 1 (ZO-1, 1:200, Abcam), (4) rabbit anti-AQP4 (1:200, Sigma-Aldrich), (5) mouse anti-glial fibrillary acidic protein (GFAP, 1:200, Santa Cruz), (6) rabbit anti-excitatory amino acid transporter 2 (EAAT2, 1:50, Abcam), (7) rabbit anti-ionized calcium-binding adapter molecule 1 (Iba-1, 1:200, Wako), (8) mouse anti-cluster of differentiation 68 (CD68, 1:200, Immunoway), (9) rat anti-F4/80 (1:200, Abcam) (10) rat anti-lymphocyte antigen 6 complex locus G6D (Ly6G, 1:400, Abcam), (11) goat anti-myelin basic protein (MBP, 1:200, Dako), (12) rabbit anti-neurofilament (NF, 1:400, Sigma-Aldrich), and (13) rabbit anti-oligodendrocyte transcription factor 2 (Olig2, 1:200, Millipore). Sections were then incubated with the appropriate Alexa-Fluor-conjugated secondary antibodies (Thermo Fisher Scientific, USA) at room temperature for 1 h. Neuronal cells were visualized with blue fluorescent Nissl stain (NeuroTrace, Thermo Fisher Scientific, USA). Sections were counterstained with DAPI and mounted with antifade reagent (Thermo Fisher Scientific, USA). Selected sections were stained with hematoxylin and eosin (H&E) and Luxol fast blue using standard procedures.

### Image Processing and Quantification

Measurements of immunofluorescent intensities were performed on eight rostral-to-caudal alternate cross sections of cervical spinal cord. The sampling areas include the white matter in the ventrolateral spinal cord and the gray matter around the central canal of the spinal cord. All signals were captured with the same microscope (Nikon Eclipse Ni) and digitized with SPOT software 5.0 (Diagnostic Instruments, Inc.) in identical settings. Signal intensities were quantified with ImageJ software (Wayne Rasband, NIH, USA).

### Statistical Analyses

Differences between groups were compared by one-way ANOVA followed by Tukey–Kramer *post hoc* test. Data are shown as mean ± SEM. Levels of significance are indicated with *p < 0.05, **p < 0.01, and ***p < 0.001. Calculation was performed using IBM SPSS Statistics Version 24 software.

## Results

### Human IgG Infiltration to Spinal Cord Without Complement Activation in Mice With Breached BBB

Western blot performed on the purified IgG_(Healthy)_, IgG_(AQP4−)_, and IgG_(AQP4+)_ validated the presence of human IgG with heavy and light chains in the samples (Figure 1B). Immunostaining revealed continuous and linear expression of the tight junction protein ZO-1 in blood vessels of the spinal cord parenchyma in normal mice, but punctate and discontinuous ZO-1 staining in mice which received pretreatment with CFA and PTx (Figure 1C, upper panel). Human IgG immunoreactivity in spinal cord parenchyma was observed in mice with pretreatment with CFA and PTx, but not in those without the pretreatment (Figure 1C, lower panel). We found the same amplitude of human IgG infiltration in the spinal cord of mice which received IgG_(AQP4+)_, IgG_(AQP4−)_, and IgG_(Healthy)_, as long as CFA and PTx were administered to breach the BBB (Figure S1 in Supplementary Material). No C5b-9 immunoreactivity was observed in IgG_(Healthy)_, IgG_(AQP4−)_, or inIgG_(AQP4+)_ mice. Spleens from EAE mice immunized with MOG_35–55_ peptide were used as a positive staining control (Figure 1D). These results confirmed that purified human IgG in the peripheral blood of mice crossed the breached BBB and infiltrated to the spinal cord parenchyma without complement activation.

### AQP4–IgG Causes Loss of AQP4 and GFAP in Spinal Cord of Mice

**Figure 2:**
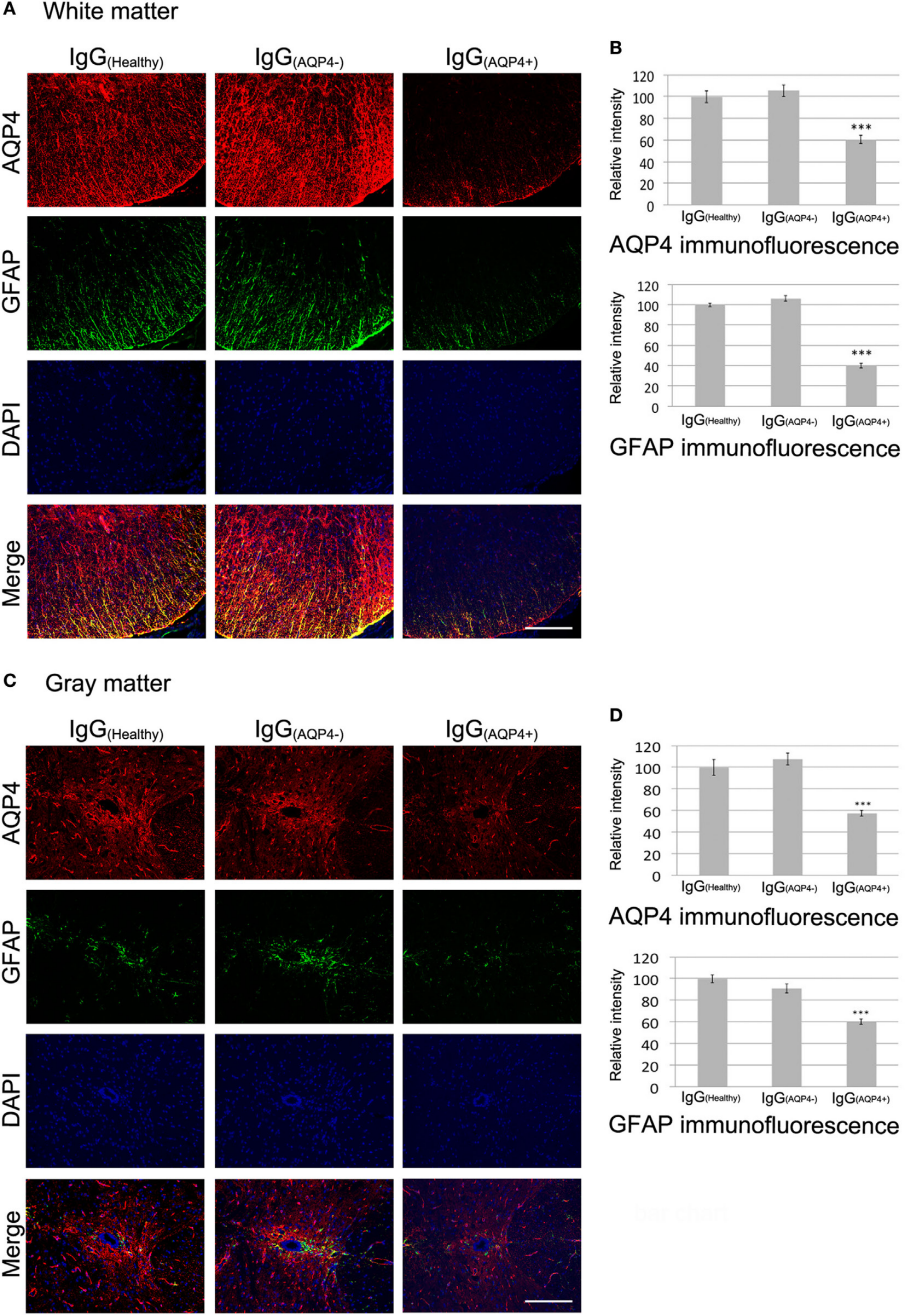
IgG_(AQP4+)_ causes AQP4 and glia fibrillary acidic protein (GFAP) loss in the spinal cord of mice. Pictures are representative photomicrographs of cross sections showing the white matter of the ventrolateral spinal cord or the gray matter around the spinal cord central canal. **(A,C)** Marked loss of immunoreactivities for AQP4 and GFAP in spinal cord white **(A)** and gray matter **(C)** of IgG_(AQP4+)_ mice, compared to IgG_(Healthy)_, and IgG_(AQP4−)_ mice. **(B,D)** Confirmation of AQP4 and GFAP loss in spinal cord white **(B)** and gray matter **(D)** of IgG_(AQP4+)_ mice (*n* = 5), compared to IgG_(Healthy)_ (*n* = 5) and IgG_(AQP4−)_ (*n* = 5) mice by evaluation of fluorescence intensity. ***p < 0.001. Scale bars = 100 µm.

We then examined the effects of IgG_(AQP4+)_ on AQP4 and GFAP levels in the spinal cord. Co-immunostaining revealed marked decrease in AQP4 and GFAP levels in the spinal cord white matter of IgG_(AQP4+)_ mice compared to IgG_(Healthy)_ and IgG_(AQP4−)_ mice. Merge of the images showed co-localized loss of AQP4 and GFAP immunoreactivities in IgG_(AQP4+)_ mice compared to the controls (Figure 2A). Significant decrease in AQP4 and GFAP levels was confirmed by evaluation of the immunofluorescence intensities (*p* < 0.001) (Figure 2B). In the gray matter, strong AQP4 and GFAP immunoreactivities were observed in the region around the spinal cord central canal in IgG_(Healthy)_ and IgG_(AQP4−)_ mice, whereas the immunoreactivities were markedly decreased in IgG_(AQP4+)_ mice. Merge of the images revealed co-localized loss of AQP4 and GFAP immunoreactivities in IgG_(AQP4+)_ mice (Figure 2C). Significant decrease in AQP4 and GFAP levels were confirmed by evaluation of the immunofluorescence intensities (*p* < 0.001, Figure 2D). There was no observable difference in AQP4 and GFAP immunoreactivities in mice which received PBS compared to those which received IgG_(Healthy)_ or IgG_(AQP4−)_ (Figure S2 in Supplementary Material). The decrease in AQP4 and GFAP levels shown by immunofluorescence was confirmed using immunoperoxidase staining (Figure S3 in Supplementary Material).

### AQP4–IgG Leads to Loss of EAAT2 in Spinal Cord of Mice

**Figure 3:**
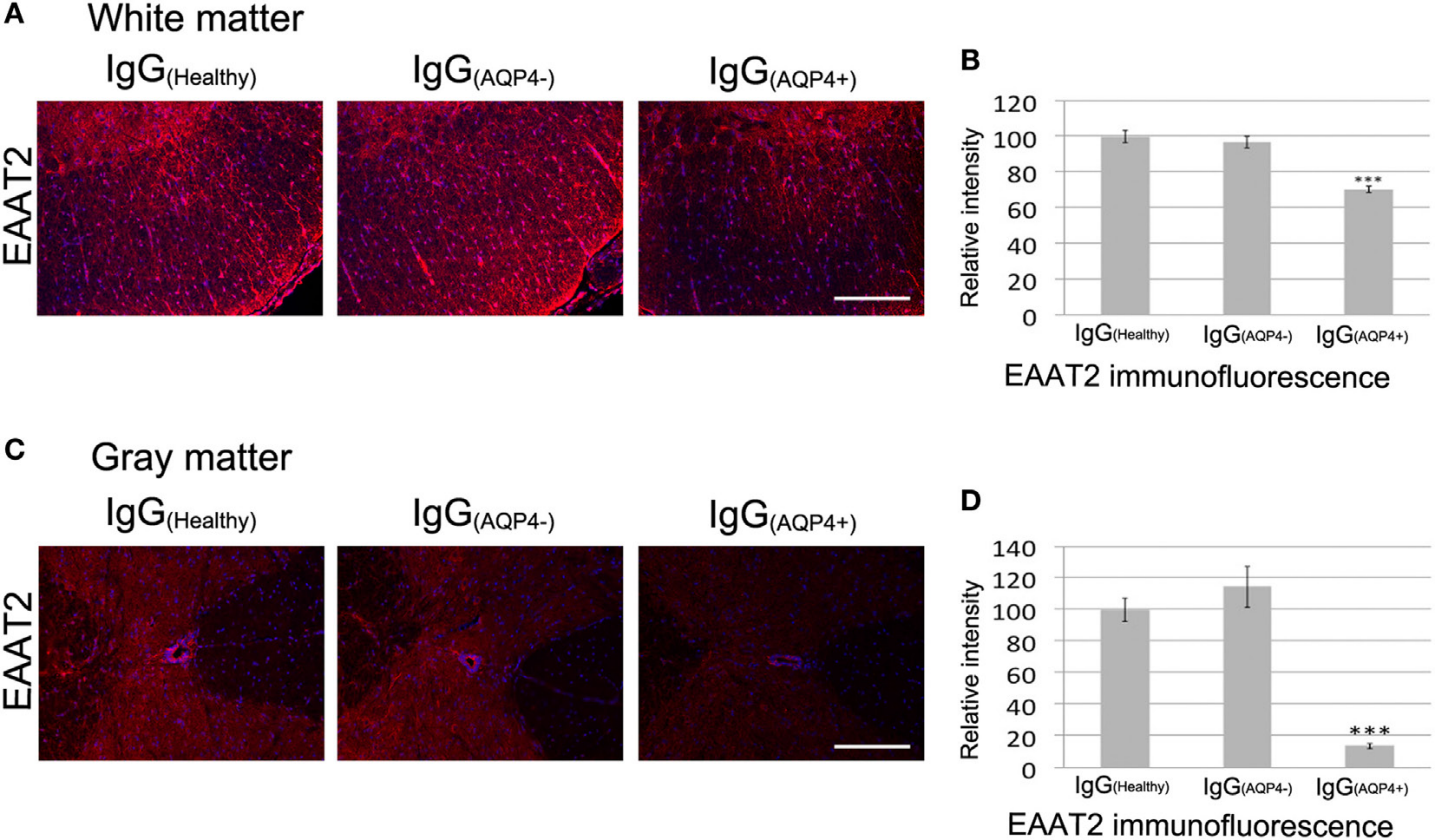
IgG_(AQP4+)_ leads to loss of the glutamate transporter excitatory amino acid transporter 2 (EAAT2) in the spinal cord of mice. **(A,C)** Marked loss of immunoreactivities for EAAT2 in spinal cord white **(A)** and gray matter **(C)** of IgG_(AQP4+)_ mice, compared to IgG_(Healthy)_ and IgG_(AQP4−)_ mice. **(B,D)** Confirmation of EAAT2 loss in spinal cord white **(B)** and gray matter **(D)** of IgG_(AQP4+)_ mice (*n* = 5) compared to IgG_(Healthy)_ (*n* = 5) and IgG_(AQP4−)_ (*n* = 5) mice by evaluation of fluorescence intensity. ***p < 0.001. Scale bars = 100 µm.

Binding of AQP4–IgG to AQP4 in astrocyte membrane triggers co-endocytosis of AQP4 and the glutamate transporter EAAT2, causing glutamate excitotoxicity (16). IHC revealed a marked decrease in EAAT2 staining in the white and gray matter of spinal cord of IgG_(AQP4+)_ mice compared to IgG_(Healthy)_ and IgG_(AQP4−)_ mice (Figures 3A,C). Evaluation of EAAT2 immunofluorescence intensities confirmed the decrease in IgG_(AQP4+)_ mice compared to controls (*p* < 0.001, Figures 3B,D). These findings suggest that IgG_(AQP4+)_ causes loss of EAAT2, which may lead to glutamate excitotoxicity in the spinal cord.

### AQP4–IgG Triggers Marked Microglial Activation, Macrophage Activation, and Mild Neutrophil Infiltration

**Figure 4:**
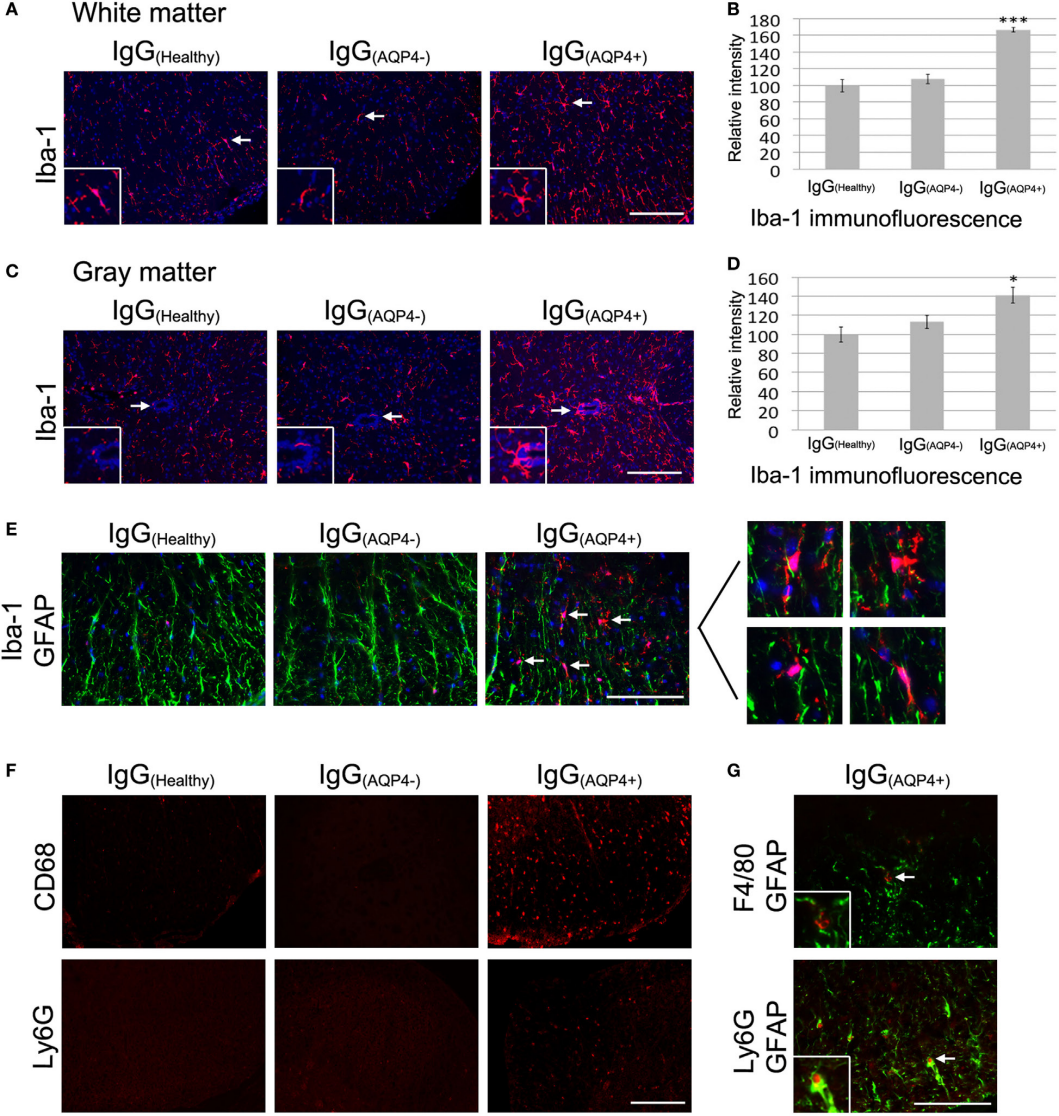
IgG_(AQP4+)_ induces microglial/macrophage activation and neutrophil infiltration in the spinal cord. **(A,C)** Marked increase in Iba-1 immunoreactivity for microglia in spinal cord white **(A)** and gray matter **(C)** of IgG_(AQP4+)_ mice compared to IgG_(Healthy)_ and IgG_(AQP4−)_ mice. **(B,D)** Confirmation of Iba-1 increase in the spinal cord white **(B)** and gray matter **(D)** of IgG_(AQP4+)_ (*n* = 5) compared to IgG_(Healthy)_ (*n* = 5) and IgG_(AQP4−)_ (*n* = 5) mice by evaluation of fluorescence intensity. ***p < 0.001; *p < 0.05. **(E)** Co-immunostaining of Iba-1 and glia fibrillary acidic protein (GFAP) revealed proximity of activated microglia/macrophages to astrocytes in the spinal cord of IgG_(AQP4+)_ mice (arrows), which was not observed in IgG_(Healthy)_ and IgG_(AQP4−)_ mice. **(F)** Cluster of differentiation 68 immunoreactivity for macrophages was observed in spinal cord of IgG_(AQP4+)_ mice but not in IgG_(Healthy)_ and IgG_(AQP4−)_ mice (upper panel). Ly6G immunoreactivity for neutrophils was observed in spinal cord of IgG_(AQP4+)_ mice, but not in IgG_(Healthy)_ and IgG_(AQP4−)_ mice (lower panel). **(G)** Co-immunostaining of F4/80 and GFAP (upper panel) and Ly6G and GFAP (lower panel) revealed proximity of macrophages and neutrophils to astrocytes (arrows) in spinal cord of IgG_(AQP4+)_ mice. Scale bars = 100 µm.

Immunofluorescence revealed a marked increase in Iba-1 staining in the white and gray matter of the spinal cord of IgG_(AQP4+)_ mice compared to IgG_(Healthy)_ and IgG_(AQP4−)_ mice, and increased number and length of microglial processes in IgG_(AQP4+)_ mice compared to the controls (Figures 4A,B). Evaluation of immunofluorescence intensities confirmed significant Iba-1 increase in IgG_(AQP4+)_ mice compared to the controls (*p* < 0.001 in white matter, *p* < 0.05 in gray matter, Figures 4C,D). Co-immunostaining of Iba-1 and GFAP revealed that activated microglia/macrophages were in close proximity to astrocytes in the spinal cord of IgG_(AQP4+)_ mice (Figure 4E). Immunofluorescence revealed CD68 and Ly6G fluorescence signals in the spinal cord of IgG_(AQP4+)_ mice, which were not observed in the spinal cord of IgG_(Healthy)_ and IgG_(AQP4−)_ mice (Figure 4F). Spleens from EAE mice immunized with MOG_35–55_ peptide were used as a positive immunostaining control (data not shown). Further co-immunostaining of F4/80 or Ly6G with GFAP revealed that macrophages and neutrophils were in close proximity to astrocytes in the spinal cord of IgG_(AQP4+)_ mice (Figure 4G). The marked increases in Iba-1 and CD68 immunoreactivities with infrequent F4/80 immunoreactivity suggested that the macrophages found in the cord of IgG_(AQP4+)_ mice were predominantly derived from resident microglial cells. H&E staining revealed inflammatory cell infiltration into the spinal cord parenchyma of IgG_(AQP4+)_ mice, which was not observed in IgG_(Healthy)_ and IgG_(AQP4−)_ mice (Figures S4A–E in Supplementary Material). Higher magnification showed the presence of polymorphonuclear leukocytes in close proximity to neurons in the cord of IgG_(AQP4+)_ mice (Figure S4F in Supplementary Material).

These findings suggest that AQP4–IgG triggers marked microglia activation and proliferation, macrophage activation, mild inflammatory cells (macrophage and neutrophil) infiltration into spinal cord parenchyma, and cellular interaction between microglia/macrophage/neutrophil and astrocyte in the cord after passive transfer of IgG_(AQP4+)_ into mice with breached BBB.

### AQP4–IgG-Induced Effects on Glial and Immune Cells Are Associated With Demyelination and Axonal Injuries/Loss, but Not Oligodendrocyte or Neuronal Loss

**Figure 5:**
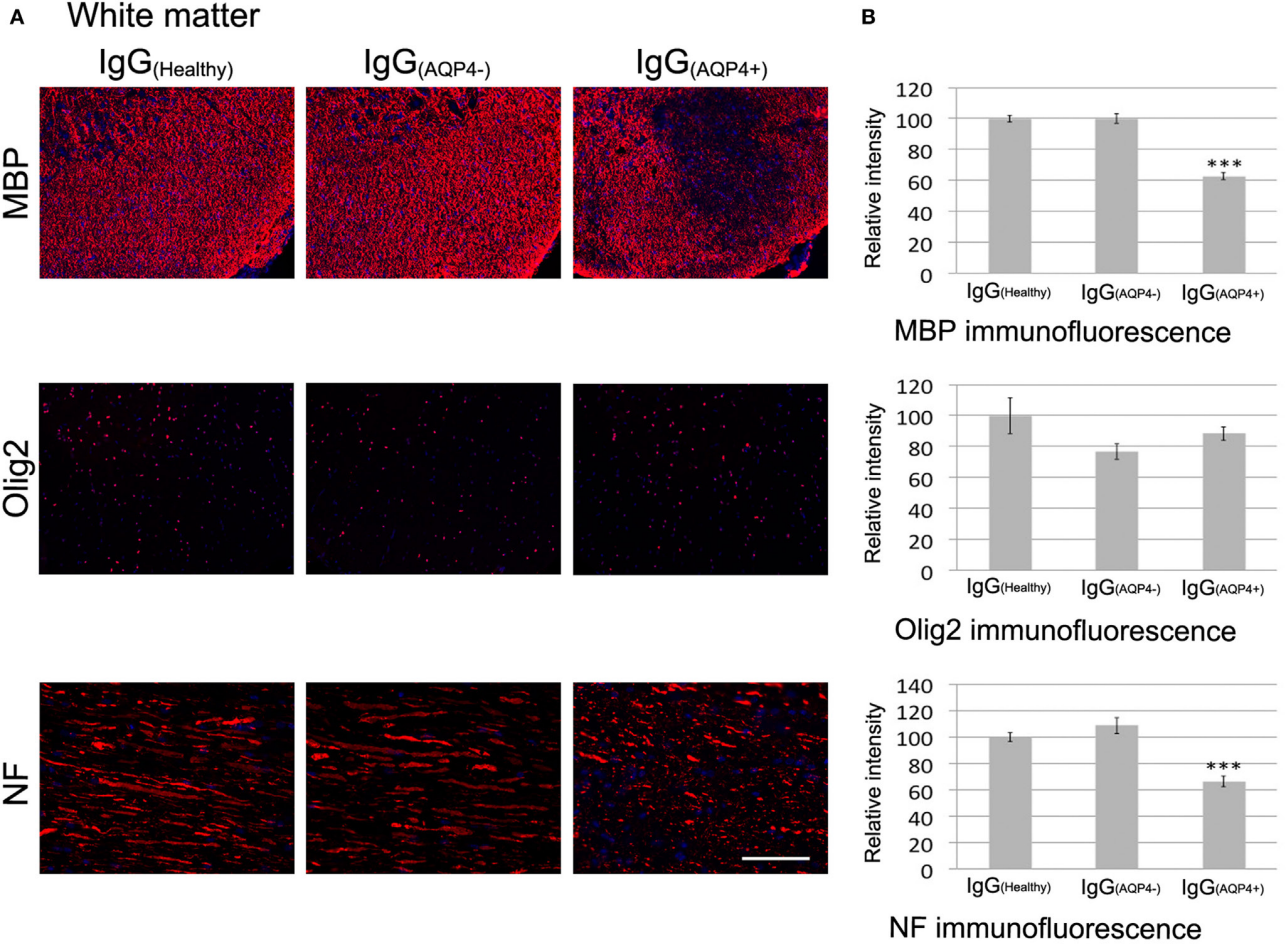

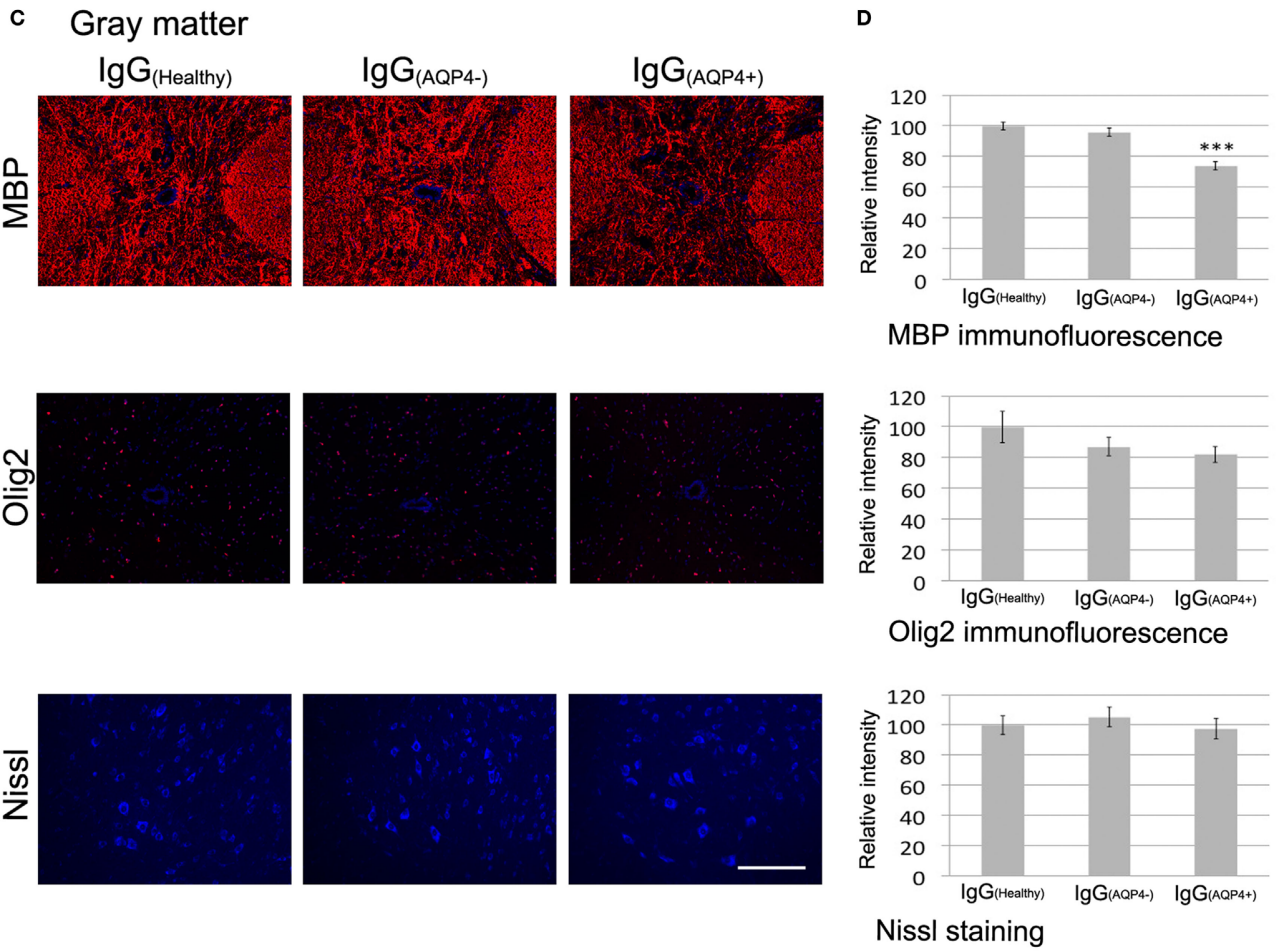
Passive transfer of IgG_(AQP4+)_ is associated with demyelination and axonal injuries/loss, but not neuronal loss, in the spinal cord of mice. **(A)** Immunofluorescence for myelin basic protein (MBP) (upper panel) revealed patchy loss of MBP immunoreactivity suggestive of demyelination in spinal cord white matter of IgG_(AQP4+)_ mice, compared to IgG_(Healthy)_, and IgG_(AQP4−)_ mice. Immunostaining for Olig2 (middle panel) showed no loss of oligodendrocytes in IgG_(AQP4+)_ mice compared to IgG_(Healthy)_ and IgG_(AQP4−)_ mice. Immunofluorescence for neurofilament (NF) of horizontal spinal cord section (lower panel) revealed axonal fragmentation and loss in white matter of IgG_(AQP4+)_ mice compared to IgG_(Healthy)_ and IgG_(AQP4−)_ mice. **(B)** Evaluations of fluorescence intensities for MBP, Olig2, and NF in the spinal cord white matter of IgG_(AQP4+)_ (*n* = 5), IgG_(Healthy)_ (*n* = 5), and IgG_(AQP4−)_ (*n* = 5) mice. ***p < 0.001. **(C)** Immunofluorescence for MBP (upper panel) revealed marked loss of MBP immunoreactivity suggestive of demyelination in spinal cord gray matter of IgG_(AQP4+)_ mice compared to IgG_(Healthy)_ and IgG_(AQP4−)_ mice. Immunostaining for Olig2 (middle panel) showed no loss of oligodendrocytes in IgG_(AQP4+)_ mice compared to IgG_(Healthy)_ and IgG_(AQP4−)_ mice. Fluorescence Nissl staining (lower panel) showed no loss of spinal motoneurons in IgG_(AQP4+)_ mice compared to IgG_(Healthy)_ and IgG_(AQP4−)_ mice. **(D)** Evaluations of fluorescence intensities for MBP, Olig2, and Nissl staining in the spinal cord gray matter of IgG_(AQP4+)_ (*n* = 5), IgG_(Healthy)_ (*n* = 5), and IgG_(AQP4−)_ (*n* = 5) mice. ***p < 0.001. Scale bars = 100 µm.

We further investigated whether IgG_(AQP4−)_-induced astrocytopathy and neuroinflammation affected the integrity of myelin, axons, and neurons in the cord of mice. In the white matter, immunofluorescence of MBP demonstrated marked and patchy loss of immunoreactivity in IgG_(AQP4+)_ mice compared to IgG_(Healthy)_ and IgG_(AQP4−)_ mice. Evaluation of immunofluorescence intensities revealed a significant decrease in IgG_(AQP4+)_ mice compared to controls (*p* < 0.001). Patchy demyelination suggested by loss of fluorescent signal for MBP in IgG_(AQP4+)_ mice was confirmed by Luxol fast blue staining (Figure S5 in Supplementary Material). However, we did not observe any significant difference in the immunofluorescence intensity of the oligodendrocyte marker Olig2 between the three groups. Immunofluorescence for NF on transverse spinal cord sections revealed fragmented fluorescent signal and significant decrease of fluorescence intensity in IgG_(AQP4+)_ mice compared to controls (*p* < 0.001), suggestive of axonal fragmentation, and loss in IgG_(AQP4+)_ mice (Figures 5A,B).

Similarly, in spinal cord gray matter, immunofluorescence of MBP demonstrated patchy loss of immunoreactivity in IgG_(AQP4+)_ mice compared to IgG_(Healthy)_ and IgG_(AQP4−)_ mice. Evaluation of immunofluorescence intensities revealed a significant decrease in IgG_(AQP4+)_ mice compared to controls (*p* < 0.001). Immunofluorescence of Olig2 for oligodendrocytes showed no significant difference in immunoreactivities between the three groups. Fluorescence Nissl staining for spinal motoneurons showed no significant difference in immunoreactivities between the three groups (Figures 5C,D).

### AQP4–IgG-Induced Spinal Cord Pathologies Are Associated With Subtle Motor Impairments

To evaluate the effects of these IgG_(AQP4+)_-induced cord pathologies on motor function of the mice, we used EAE score to assess the motor function daily and beam walking test to detect subtle motor abnormalities after IgG transfer. EAE score showed that mice in all three groups had no clinical evidence of motor weakness during the course of IgG transfer (score 0 from day 0 to day 8, data not shown).

**Figure 6:**
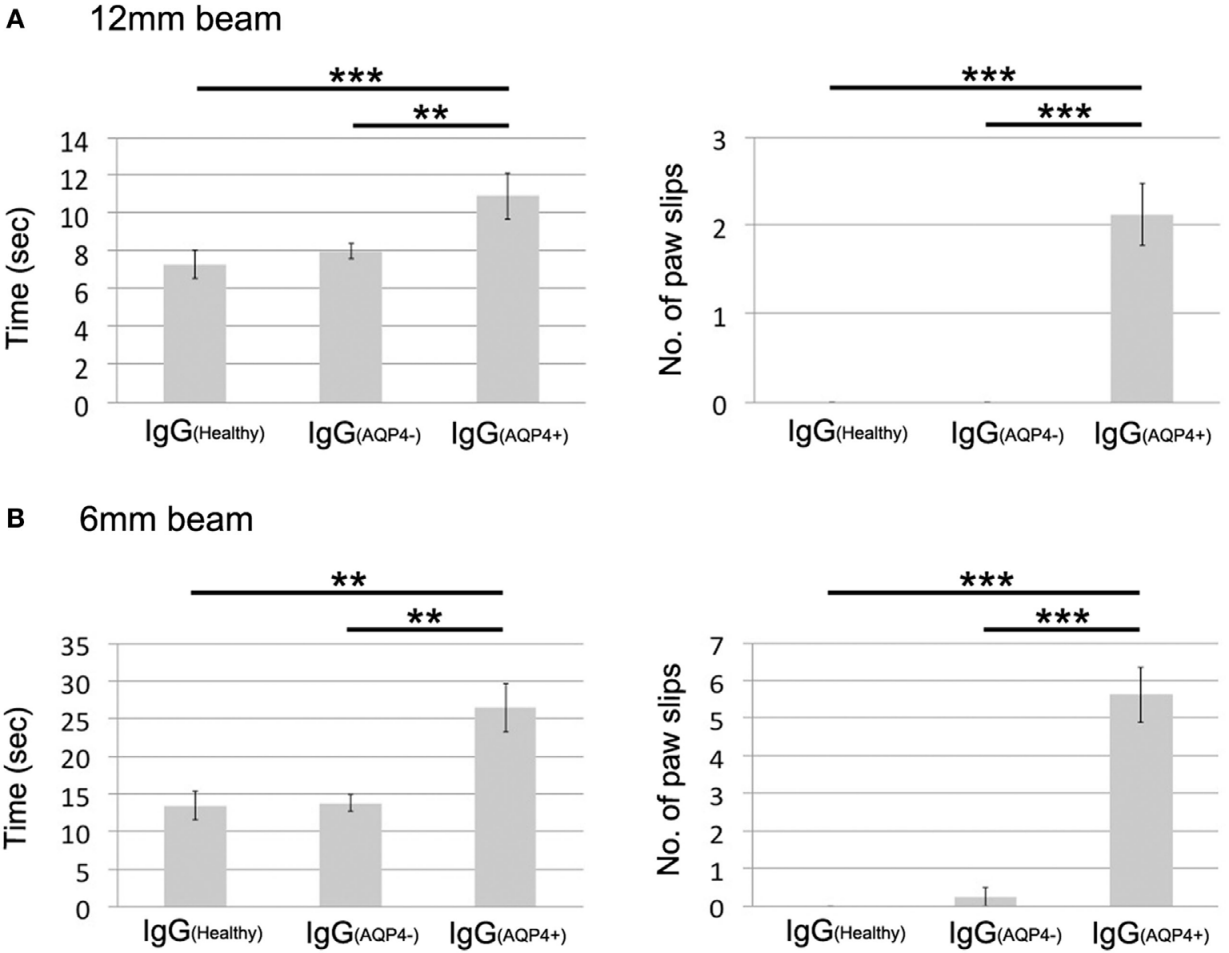
Subtle motor impairments of mice which received IgG_(AQP4+)_ detected by beam walking test. **(A)** Beam walking test measured time required to cross the beam and number of paw slips during walking on a 12 mm wide and 80 cm long beam. IgG_(AQP4+)_ mice (*n* = 8) required significantly longer time and had more slips than IgG_(Healthy)_ (*n* = 8) and IgG_(AQP4−)_ (*n* = 8) mice. **(B)** Similar results were observed in beam walking test on a narrower 6 mm wide and 80 cm long beam. **p < 0.01; ***p < 0.001.

On beam walking test, IgG_(AQP4+)_ mice required significantly longer time to cross the 12 mm-wide beam compared to IgG_(Healthy)_ (*p* < 0.001) and IgG_(AQP4−)_ (*p* < 0.01) mice. IgG_(AQP4+)_ mice had significantly more paw slips during walking on the beam than controls (*p* < 0.001, Figure 6A; Videos S1–S3 in Supplementary Material). Similar findings were observed with walking test on the narrower 6 mm-wide beam that allowed detection of even more subtle motor impairments. IgG_(AQP4+)_ mice required significantly longer time to cross the 6 mm-wide beam compared to IgG_(Healthy)_ and IgG_(AQP4−)_ mice (*p* < 0.01), and IgG_(AQP4+)_ mice had more slips during walking on the 6 mm-wide beam than controls (*p* < 0.001, Figure 6B; Videos S4–S6 in Supplementary Material).

## Discussion

We observed marked loss of AQP4 and GFAP in IgG_(AQP4+)_ mice, supporting that AQP4–IgG leads to astrocyte cytotoxicity in the absence of complement activation. It has been shown that AQP4–IgG-induced astrocytic activation leads to increased production and secretion of proinflammatory cytokines, chemokines, and other mediators of inflammation and oxidative stress (24). These inflammatory mediators and chemokines may contribute to the marked microglial activation and proliferation, macrophage activation and mild neutrophil infiltration observed in IgG_(AQP4+)_ mice in our study. This is consistent with previous findings from others. Intravitreal injection of AQP4–IgG causes complement-independent increase in the number of microglia throughout the inner retinal layers and loss of retinal ganglion cells in rats (25). Loss of AQP4, EAAT2, and GFAP is observed in the Iba-1-positive perivascular areas of the optic tract in a rat NMOSD model with extensive astrocytopathy triggered by recombinant anti-AQP4 monoclonal antibody (26). Prominent microglial activation is observed in the spinal cord lesion of patients with NMOSD (27, 28). Microglial activation is not observed in our controls, including IgG_(Healthy)_ and IgG_(AQP4−)_ mice, with CFA and PTx administered. Hence, microglial activation observed in IgG_(AQP4+)_ mice in our study is not triggered by the bacterial proteins.

Without complement activation, we propose that astrocyte loss may be mediated by antibody-dependent cell-mediated cytotoxicity (ADCC) predominantly *via* activated microglia and macrophages (mostly derived from resident microglia, and fewer infiltrated from peripheral blood) and to a lesser extent *via* infiltrated neutrophils. Human microglia express Fc receptors, including FcγRI, FcγRII, and FcγRIII, for IgG binding (29). Fc receptors on activated microglia and macrophages can bind to the Fc portion of astrocytic AQP4-linked AQP4–IgG to trigger release of cytotoxic compounds and phagocytosis of astrocytes. Similarly, infiltrated neutrophils also express Fc receptor and can contribute to astrocyte loss *via* ADCC. These mechanisms are supported by our findings that microglia, macrophages, and neutrophils were in close proximity to astrocytes, suggestive of inflammatory cell-astrocyte interaction in the cord of IgG_(AQP4+)_ mice. It has been reported that binding of AQP4–IgG to astrocytic AQP4 increases BBB permeability, and causes natural killer cell degranulation and astrocyte cytotoxicity *in vitro* (5). Injection of human AQP4–IgG and natural killer cells to mouse brain produces NMOSD-like lesions featuring AQP4 and GFAP loss (30). Mice treated with AQP4–IgG incapable of ADCC activation and human complement have attenuated NMOSD-like pathologies (31). These findings support that ADCC contributes to AQP4–IgG-induced astrocytic injury and cytotoxicity in NMOSD lesions. Additionally, chronic intrathecal infusion of AQP4–IgG to rats results in astrocyte damage associated with myelin alternation, loss of axons and oligodendrocytes, and motor impairment in the absence of complement activation and immune cell infiltration (32). Direct AQP4–IgG-mediated astrocytopathy, without involvement of immune cells and complements, may lead to astrocytic loss as observed in our model.

Other mechanisms involved in AQP4–IgG-induced astrocyte cytotoxicity may include glutamate excitotoxicity as astrocytes also express glutamate receptors. AQP4 is physiologically coupled with the glutamate transporter EAAT2 in astrocytic membrane. Without complements, cultured astrocytes exposed to NMOSD patient serum are observed to have AQP4 endocytosis with concomitant loss of EAAT2 and reduction of glutamate reuptake (6). In primary culture of astrocytes and oligodendrocytes with prolonged exposure to IgG from AQP4–IgG-seropositive patients, the activity of glutamine synthase in astrocytes is decreased, in parallel with progressive accumulation of glutamate in the culture medium, loss of oligodendritic cell processes and oligodendrocyte death (7). Intrathecal administration of IgG from AQP4–IgG-seropositive patients to rats reduces astrocytic EAAT2 level, and induces cord lesions without complement activation (33). Internalization of membrane AQP4 upon AQP4–IgG binding requires astrocytic Fc gamma receptor, which induces co-endocytosis of EAAT2 (16). Our findings showed that IgG_(AQP4+)_ mice had marked EAAT2 loss in white and gray matter of the spinal cord, which are consistent with previous studies and suggest that AQP4–IgG-induced EAAT2 loss may contribute to NMOSD pathophysiologies *via* glutamate excitotoxicity.

We observed that IgG_(AQP4+)_ mice had patchy demyelination and widespread axonal injuries/loss, but no oligodendrocyte or neuronal cytotoxicity. Oligodendrocytes and neurons are not directly damaged by AQP4–IgG as they do not express AQP4 (11). The observed demyelination in IgG_(AQP4+)_ mice is likely due to activated microglia and macrophages, and also possibly oligodendrocyte dysfunction secondary to glutamate excitotoxicity. Activated microglia and macrophages can lead to demyelination directly *via* phagocytosis as documented in active multiple sclerosis lesions (34). Additionally, oligodendrocytes express glutamate receptors and are susceptible to glutamate excitotoxicity (35). Demyelination found in IgG_(AQP4+)_ mice in our study may be related to impaired myelinating function of oligodendrocytes due to glutamate excitotoxicity secondary to loss of astrocytic EAAT2. The observed axonal injuries/loss may be due to activated microglia/macrophages which directly damage axons especially in demyelinated areas, as well as glutamate excitotoxicity to neurons leading to secondary axonal degeneration. In particular, we observed that EAAT2 loss was more severe in spinal cord gray matter where neuronal cell bodies are located. In addition, lack of trophic support from astrocytes may result in myelin and axonal injuries (36–38).

Our results revealed that IgG_(AQP4+)_ mice had subtle motor impairments detected on beam walking test, which were not shown on EAE score. We observed that IgG_(AQP4+)_ mice had hind leg swelling which was not found in control mice, suggesting the possibility of AQP4–IgG-induced myositis in hind leg regions of immunization. We cannot exclude the possibility that this may contribute to the subtle motor impairments detected on beam walking test. Autoimmune AQP4 myopathy with recurrent hyperCKemia has been reported in NMOSD patients (39). The pathology of muscle caused by AQP4–IgG is beyond the aim of the current study. It will be important to investigate whether AQP4–IgG causes elevation in serum creatine kinase level and sacrolemmal damage in our model. The motor slowing and impairment of fine motor coordination can be explained by the observed demyelination and axonal injuries. In patients with NMOSD, myelin water imaging identifies widespread demyelination in the normal-appearing white matter, suggestive of subclinical myelin loss (40). Prior to thinning of retinal nerve fiber layer, subclinical primary retinal pathology and optic neuropathy have been reported in NMOSD patients (41, 42). Our findings implicate that the complement-independent pathologies induced by AQP4–IgG may cause subtle abnormalities in motor function, and these pathologies may explain the mild and asymptomatic myelitis observed in some NMOSD patients. In addition, these pathologies, including astrocytopathy and neuroinflammation, may lead to further breakdown of BBB to facilitate entry of AQP4–IgG and complements to the CNS, which then trigger severe neuroinflammation and irreversible CNS damage such as necrosis *via* complement activation. As mentioned, the normal CNS lacks the initiator of complement cascade, C1q; hence initial access of AQP4–IgG to the CNS and binding to astrocytic AQP4 do not activate complements. Complement-independent pathophysiological mechanisms likely contribute to early development of NMOSD lesion.

Previous *in vivo* studies on NMOSD largely relied on direct delivery of AQP4–IgG to the CNS of animals (10–12, 32, 33). In contrast, AQP4–IgG is detected in the peripheral blood in the majority of NMOSD patients. The mechanism of how peripheral AQP4–IgG enters the CNS in NMOSD is unclear. Systemic infections trigger inflammatory responses and breakdown of BBB *via* increased levels of circulating proinflammatory cytokines (43, 44), which can result in AQP4–IgG entry into the CNS followed by development of acute attack of NMOSD. An early report indicated viral infections prior to attack in 25% of the NMOSD patients (45). These proinflammatory cytokines include IL-1β, IL-6, and TNFα, among which IL-6 is particularly relevant in NMOSD as it has been reported that IL-6 can drive secretion of AQP4–IgG by plasmablasts which are increased in NMOSD patients (46). Our experiments partially recapitulate the development of human NMOSD lesions by intraperitoneal transfer of AQP4–IgG to mice with BBB breached by immunization with bacterial proteins. This approach allows rapid absorption of human IgG to the animal’s peripheral blood, followed by entry of human IgG into the animal’s CNS *via* the breached BBB.

One of the limitations of our study is that we only examine the effects of human AQP4–IgG in the spinal cord of mice. The possibility that the subtle motor impairments detected in IgG_(AQP4+)_ mice is also related to brain pathologies cannot be excluded. Further experiments are currently underway in our laboratory to study the complement-independent effects of human AQP4–IgG on different areas of the mouse CNS, including the brain and optic nerve. In addition, to understand the correlation of human AQP4–IgG and mouse pathologies, further experiments are needed to examine the time course of human IgG infiltration and its relationship with AQP4 and GFAP loss in the mouse CNS.

In conclusion, we found that passive transfer of purified IgG from AQP4–IgG-seropositive NMOSD patients intraperitoneally to mice with breached BBB caused complement-independent spinal cord pathologies, including AQP4 and astrocyte loss, neuroinflammation, EAAT2 loss, demyelination, and axonal injuries/loss which were associated with subtle motor impairments. Our findings suggest that complement-independent pathologies triggered by AQP4–IgG-astrocytic AQP4 binding may underlie clinically mild or asymptomatic myelitis observed in some NMOSD patients, and may precede development of severe myelitis characterized by complement-dependent necrotic inflammation. Understanding of these pathophysiologies provides insights into potential targets for developing novel therapeutics for NMOSD, such as glutamate antagonists, and inhibitors of microglial activation and ADCC.

## Ethics Statement

All procedures were approved by the Committee on the Use of Live Animals in Teaching and Research of The University of Hong Kong. Informed consent for study was obtained from all study subjects. The use of human material for study was approved by the Institutional Review Board of the University of Hong Kong/Hospital Authority Hong Kong West Cluster (HKU/HA HKW IRB).

## Author Contributions

L-WY conceived, designed, and performed all experiments and drafted the manuscript. OM involved in beam walking test. RN gave suggestions on experimental design. JK worked on IgG preparation and purification. K-HC conceived the study, arranged patients for blood taking, and revised the manuscript. All authors agreed the data and the final manuscript.

## Conflict of Interest Statement

The authors declare that the research was conducted in the absence of any commercial or financial relationships that could be construed as a potential conflict of interest.

## Supplementary Material

Figure S1 Immunofluorescence of human IgG revealed human IgG infiltration into the spinal cord parenchyma of mice which have received IgG_(AQP4−)_ and IgG_(Healthy)_ after blood–brain barrier breached by CFA and pertussis toxin (PTx) treatment, compared to controls without CFA and PTx treatment. Scale bars = 100 µm.

Figure S2 Immunofluorescence revealed no loss of AQP4 and glia fibrillary acidic protein immunoreactivities in spinal cord white matter and gray matter of control mice which have received PBS. Scale bars = 100 µm.

Figure S3 Immunoperoxidase staining revealed marked loss of AQP4 and glia fibrillary acidic protein in the spinal cord white matter of IgG_(AQP4+)_ mice, compared to IgG_(Healthy)_ and IgG_(AQP4−)_ mice. Scale bars = 100 µm.

Figure S4 Hematoxylin and eosin staining revealed infiltration of inflammatory cells in spinal cord of IgG_(AQP4+)_ mice. **(A–C)** Spinal cord white matter of mice which have received PBS **(A)**, IgG_(Healthy)_ **(B)**, and IgG_(AQP4−)_ **(C)**. **(D)** Spinal cord white matter lesion of IgG_(AQP4+)_ mice. **(E)** Higher magnification of **(D)** showing the presence of inflammatory cells in the lesion area (arrows). **(F)** Presence of polymorphonuclear leukocytes in proximity to a neuron in the spinal cord gray matter of IgG_(AQP4+)_ mice (arrows). Scale bars = 100 µm.

Figure S5 Luxol fast blue staining revealed patchy loss of myelin in spinal cord white matter of IgG_(AQP4+)_ mice, compared to IgG_(Healthy)_ and IgG_(AQP4−)_ mice (upper panel, cross section; lower panel, horizontal section). Scale bars = 100 µm.

Videos S1–S3 Representative video clips showing the beam walking test of the mouse which have received IgG_(Healthy)_, IgG_(AQP4−)_, or IgG_(AQP4+)_ (videos 1, 2, and 3, respectively) on the 12 mm-wide beam. The mouse which have received IgG_(AQP4+)_ required longer crossing time and had more slips than those that have received IgG_(Healthy)_ or IgG_(AQP4−)_.

Videos S4–S6 Representative video clips showing the beam walking test of the mouse which have received IgG_(Healthy)_, IgG_(AQP4−)_, or IgG_(AQP4+)_ (videos 4, 5, and 6 respectively) on the 6 mm-wide beam. The mouse which have received IgG_(AQP4+)_ required longer crossing time and had more slips than those that have received IgG_(Healthy)_ or IgG_(AQP4−)_.
