# Supplementary figures and images for: Aquaporin-4 Autoantibodies From Neuromyelitis Optica Spectrum Disorder Patients Induce Complement-Independent Immunopathologies in Mice
Source: Front Immunol. 2018 Jun 25;9:1438. doi: 10.3389/fimmu.2018.01438 (PMC6026644; doi:10.3389/fimmu.2018.01438)

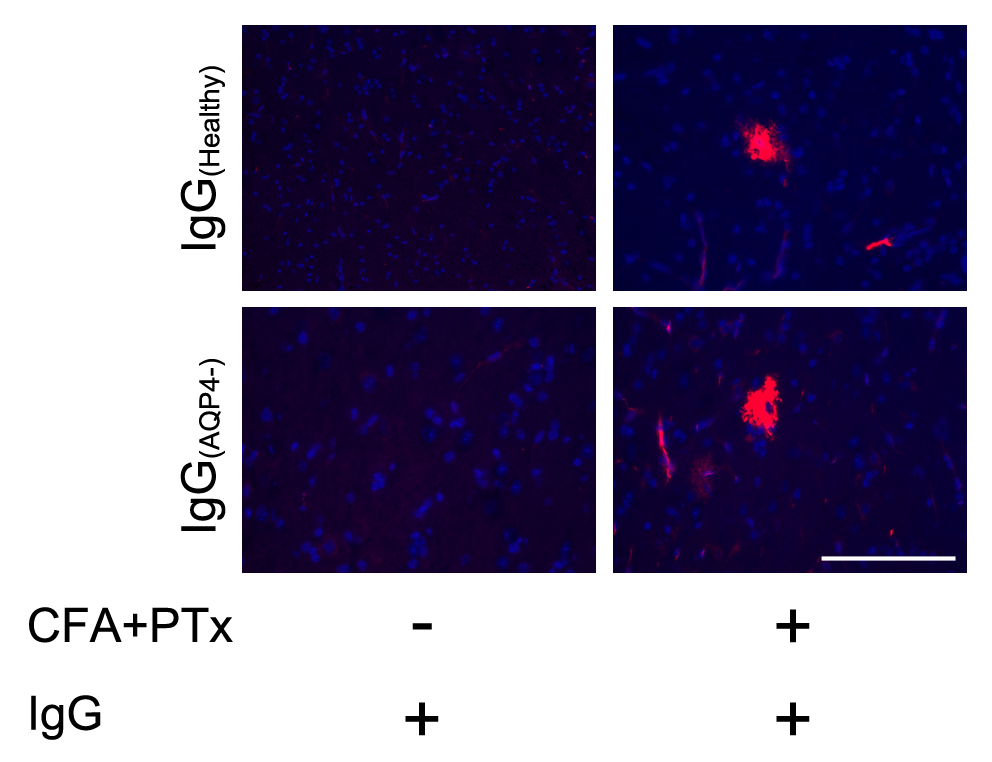

Supplement: Figure S1 — Immunofluorescence of human IgG revealed human IgG infiltration into the spinal cord parenchyma of mice which have received IgG(AQP4−) and IgG(Healthy) after blood–brain barrier breached by CFA and pertussis toxin (PTx) treatment, compared to controls without CFA and PTx treatment. Scale bars = 100 µm. [file image_1.tif]

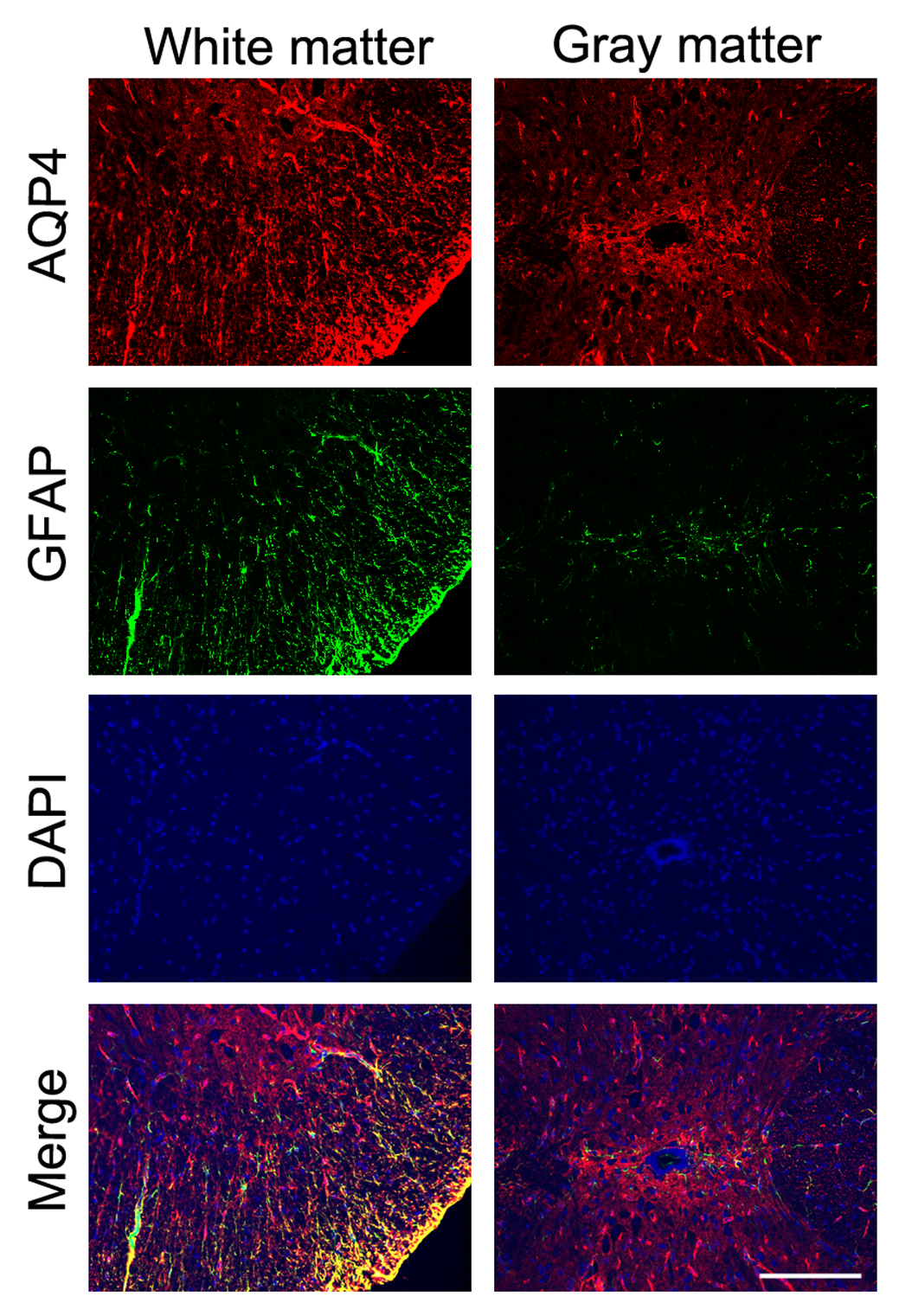

Supplement: Figure S2 — Immunofluorescence revealed no loss of AQP4 and glia fibrillary acidic protein immunoreactivities in spinal cord white matter and gray matter of control mice which have received PBS. Scale bars = 100 µm. [file image_2.tif]

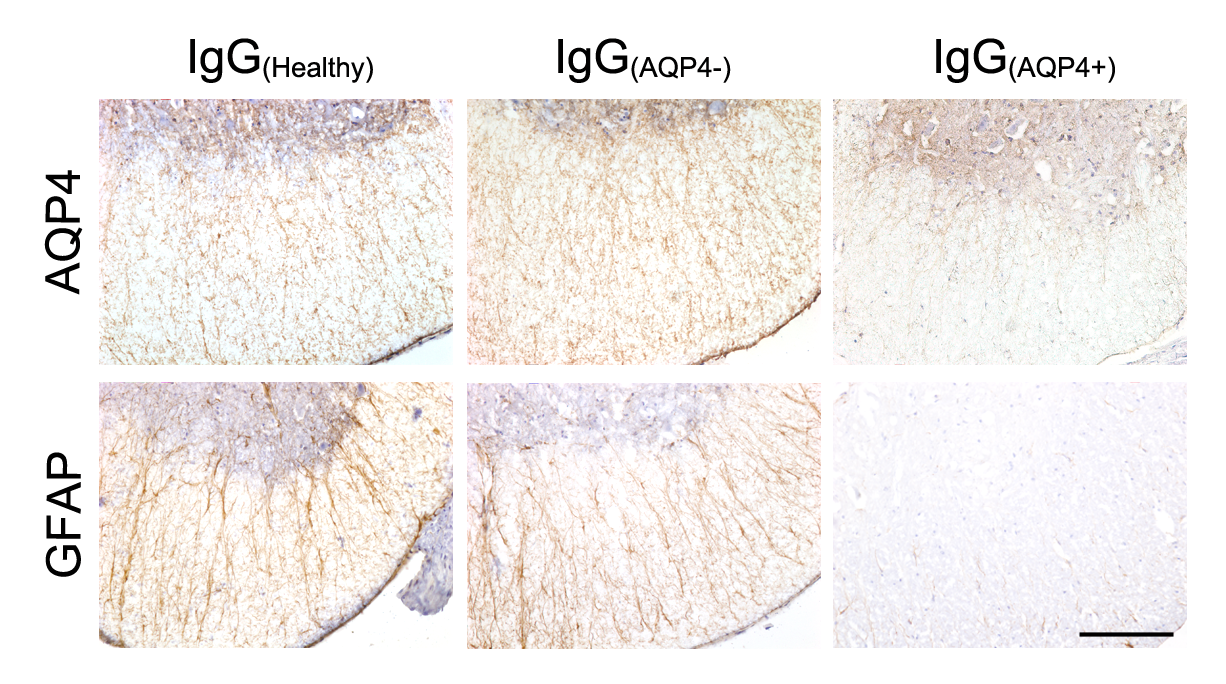

Supplement: Figure S3 — Immunoperoxidase staining revealed marked loss of AQP4 and glia fibrillary acidic protein in the spinal cord white matter of IgG(AQP4+) mice, compared to IgG(Healthy) and IgG(AQP4−) mice. Scale bars = 100 µm. [file image_3.tif]

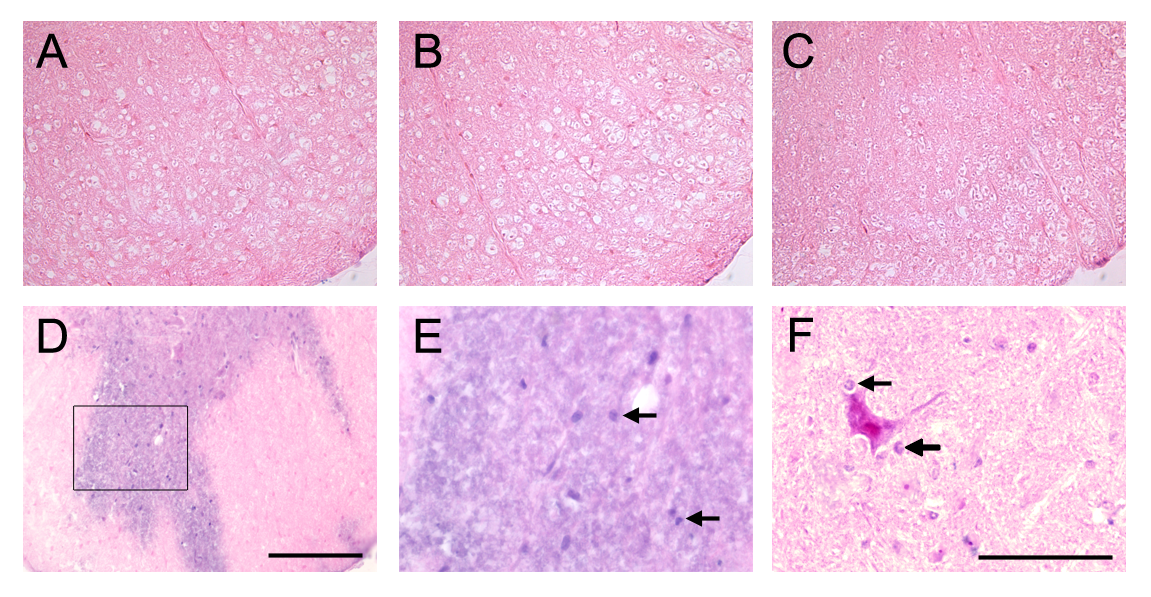

Supplement: Figure S4 — Hematoxylin and eosin staining revealed infiltration of inflammatory cells in spinal cord of IgG(AQP4+) mice. (A–C) Spinal cord white matter of mice which have received PBS (A), IgG(Healthy) (B), and IgG(AQP4−) (C). (D) Spinal cord white matter lesion of IgG(AQP4+) mice. (E) Higher magnification of (D) showing the presence of inflammatory cells in the lesion area (arrows). (F) Presence of polymorphonuclear leukocytes in proximity to a neuron in the spinal cord gray matter of IgG(AQP4+) mice (arrows). Scale bars = 100 µm. [file image_4.tif]

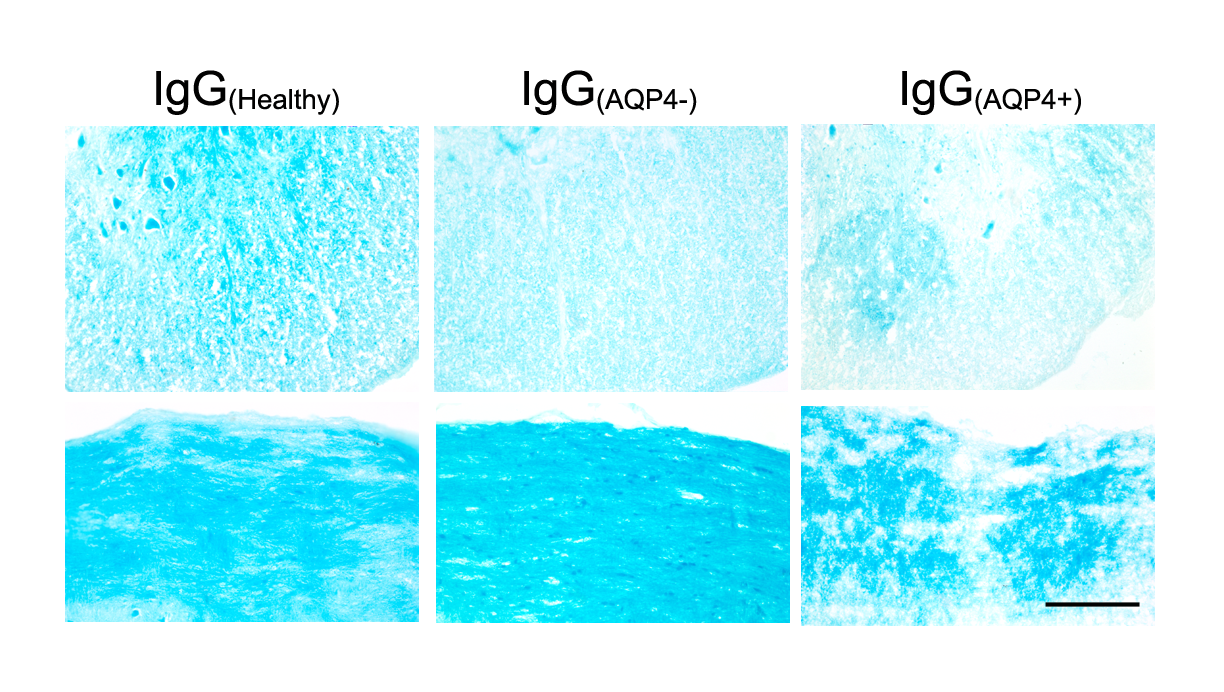

Supplement: Figure S5 — Luxol fast blue staining revealed patchy loss of myelin in spinal cord white matter of IgG(AQP4+) mice, compared to IgG(Healthy) and IgG(AQP4−) mice (upper panel, cross section; lower panel, horizontal section). Scale bars = 100 µm. [file image_5.tif]
